# Genetic Variations in *COMT* and *DRD2* Modulate Attentional Bias for Affective Facial Expressions

**DOI:** 10.1371/journal.pone.0081446

**Published:** 2013-12-02

**Authors:** Pingyuan Gong, Guomin Shen, She Li, Guoping Zhang, Hongchao Fang, Lin Lei, Peizhe Zhang, Fuchang Zhang

**Affiliations:** 1 Center for Brain and Cognitive Sciences and Department of Psychology, Peking University, Beijing, China; 2 Laboratory of Medical Molecular Biology, Henan University of Science and Technology, Luoyang, China; 3 China Academy of Corporate Governance/Business School, Nankai University, Tianjin, China; 4 Institute of Population and Health, College of Life Science, Northwest University, Xi'an, China; Xi'an Jiaotong Univesity School of Medicine, China

## Abstract

Studies have revealed that catechol-O-methyltransferase (COMT) and dopaminegic receptor2 (DRD2) modulate human attention bias for palatable food or tobacco. However, the existing evidence about the modulations of *COMT* and *DRD2* on attentional bias for facial expressions was still limited. In the study, 650 college students were genotyped with regard to *COMT* Val158Met and *DRD2* TaqI A polymorphisms, and the attentional bias for facial expressions was assessed using the spatial cueing task. The results indicated that *COMT* Val158Met underpinned the individual difference in attentional bias for negative emotional expressions (*P* = 0.03) and the Met carriers showed more engagement bias for negative expressions than the Val/Val homozygote. On the contrary, *DRD2* TaqIA underpinned the individual difference in attentional bias for positive expressions (*P* = 0.003) and individuals with TT genotype showed much more engagement bias for positive expressions than the individuals with CC genotype. Moreover, the two genes exerted significant interactions on the engagements for negative and positive expressions (*P* = 0.046, *P* = 0.005). These findings suggest that the individual differences in the attentional bias for emotional expressions are partially underpinned by the genetic polymorphisms in *COMT* and *DRD2*.

## Introduction

Attentional bias, a tendency that individuals exhibit high sensitivity and selective attention to special stimuli or relevant information [1], [2], is essential to human survival and interactions in different situations [3], [4] because this bias is involved in many cognitive and behavioral biases such as visual searching for behavioral relevant features [5], emotional response to novel visual stimuli [6], recall of threatening words [7], and drug seeking [8].

Attentional bias to special stimuli is widely varied between individuals. It has been suggested that dopamine plays a specific role in drawing attention to emotional events [9], and attentional bias to substance-related cues emerges as a result of dopaminergic activity [10]. However, the existing evidence is insufficient to understand the molecular basis of attentional bias. In the study, the main purpose is to investigate to what content of dopaminergic modulations on the individual difference in attentional bias.

Substance-related cues strongly draw individuals' attention, and they are the causes of certain behavioral disorders such as obesity [11] and drug abuse [12]. However, these cues deliver a little interpersonal information [13]. Differently, the facial expressions, as a non-verbal social communication [14], contain much social information and have great power to effect human interpersonal activities [15], [16]. Studies have indicated that the deficits in facial expressions processing are involved in the pathogenesis of many psychiatric disorders such as autism [17] and depression [18], and children with a history of physical abuse exhibit attentional bias for angry faces [19]. Therefore, to examine what content of dopaminergic modulation on attentional bias for interpersonal information, 270 facial expressions were selected as the cues of attention task in the present study.

Recent evidence has indicated that an increased dopamine activity in the prefrontal cortex enhances the attentional bias for reward-related cues such as palatable food and tobacco [10], [20], [21]. However, only a few studies have indicated that dopamine has great affects on the processing of facial expression, of which an acute dopaminergic blockade in healthy volunteers results in a transient disruption of the recognition of facial expressions of anger, whilst leaving the intact recognition for fear and disgust [22].

One of the most concerned proteins in dopamine system is Catechol-*O*-methyltransferase (COMT). Much evidence has suggested that this protein degrades catecholamines such as dopamine and norepinephrine [23] and greatly regulates the level of dopamine in brain. The *COMT* Val158Met (rs4680), a single G/A base pair substitution at codon 158, is related to the dopamine levels. Studies have indicated that Met allele of Val158Met can reduce the activity of COMT to one-quarter of what is originally encoded by Val allele [24], [25].

It was shown that *COMT* Val158Met modulates the brain activity during processing of negative stimuli, but not the stimuli with positive valence. Specifically, the Met/Met carriers, as compared to the Val/Val carriers, are more sensitive to the unpleasant stimuli [26]. Therefore, given the regulation of COMT on dopamine activity and the link between COMT activity and Val158Met variation, we predict that Val158Met is associated with attentional bias for negative expressions and the individuals with Met allele display more attentional bias for negative emotional expressions.

The actions of dopamine in brain are mediated by multiple dopaminegic receptors. Dopamine receptor D2 (DRD2) is one of the most abundant receptors in central nervous system [27]. It has been suggested that this receptor is involved in reward-related psychiatric disorders [28], [29] such as addiction and schizophrenia [30], [31]. Studies have evidenced that the density and binding site of DRD2 are influenced by several functional polymorphisms in *DRD2* [32], [33]. TaqIA (rs1800497) is the most concerned functional polymorphism in the gene. This polymorphism is related to the release of dopamine in the synaptic [34], [35], and T allele carriers are known to have a 30–40% decreased density of DRD2 [32], [34], [35]. Recent years, studies have indicated that TaqIA underlines the individual differences in work memory [36], sustained attention [37], [38], substance addiction [30], [39], and a reduced capacity in learning negative characteristics of stimuli [40]. However, the modulation of TaqIA on the attention to facial expressions, especially to the pleasure facial expressions, has not been well investigated. Here, we hypothesize that TaqIA is associated with the attentional bias for positive expressions because of the T allele carriers of this variant showing a reduced capacity for learning negative characteristics of stimuli [40].

The current study aimed to explore the modulations of *COMT* Val158Met and *DRD2* TaqIA on attentional bias for emotional expressions in a nonclinical college student sample. Moreover, although evidence has shown that the T allele of TaqIA and Met allele of *COMT* both lead to increased dopamine levels of at the synaptic clef, few studies have investigated to what extent the two genes interact to attentional bias. Guided by the previous studies, we predict that *COMT* Val158Met and *DRD2* TaqIA are associated with attentional bias for different affective facial expressions and interact to affect the bias.

## Methods

### Ethical Approval

The ethics committees of Peking University and Henan University of Science and Technology approved the study. Participants signed informed consent before taking part. The study was carried out according to the principles of the Declaration of Helsinki.

### Participants

Seven hundred right-handed participants, aging from 20 to 22, were recruited randomly from Henan University of Science and Technology in China. These subjects underwent mental health examinations by using Self-rating Depression scale [41], [42], Self-rating Anxiety scale [43], and UCLA (University of California, Los Angeles) Loneliness scale [44]. Thirty five participants, including 18 individuals with depressive symptoms (standard cut-off point of ≥50), 12 individuals with anxiety (standard cut-off point of ≥50) and 5 individuals with higher loneliness level (standard cut-off point of ≥50), were excluded from this study. Moreover, we surveyed the quantity of smoking (1.60 cigarettes) and the frequency of drinking (0.23) of each month, which indicated these subjects had no the habits of smoking and alcohol addiction. Finally, 665 unrelated Chinese Han volunteers (499 females and 166 males), with about 13 years education, were formally recruited. All subjects were Chinese Han individuals in origin by self-report. The hair follicle cells were collected after informed consents were obtained.

### Attentional bias assessment

Two hundred and seventy facial photographs, including 90 neutral expressions, 90 negative expressions (30 sad faces, 30 fearful faces and 30 angry faces) and 90 happy expressions, were selected from the Chinese Affective Picture System [45]. All the pictures were assessed on the intensity (Mean ± SD) with 9-point rating scale (1 =  most weak, 9  =  most intensive) by the designers [45]. Each emotional faces category consisted of 45 female facial photographs and 45 male facial photographs. The three photograph categories were matched in intensity (negative 5.66±0.99, neutral 5.75±0.19, happy 5.22±1.06).

The spatial cueing task was used to assess attentional bias [46], [47], [48], [49]. In the task, the participants focused on a fixation point in the center of screen. Then cue was presented, and a target was followed appearing on the left or right side of screen. Participants indicated the location of the target as quickly as possible. In the trails, the cues were valid when the cues and the targets were appeared in the same locations, otherwise the cues were invalid. Valid trails are benefit to direct attention to the cued location whereas invalid trails might promote orienting to the uncued location.

In this task, the reaction times (RTs) of valid trials are shorter than those of the invalid trials when stimulus-onset asynchrony is less than 300 ms. On the contrary, the RTs of valid trials are longer than those of the invalid trials once the stimulus-onset asynchrony (SOA) exceeds 300 ms [50], [51], [52]. The attentional bias was decomposed into cue validity, engagement and disengagement. Cue validity (RT*_invalid cue_* -RT*_valid cue_*) provides a measure of overall attention for the different cue types, of which positive scores indicate attention away valid cue whereas negative scores indicate attention toward the valid cue when SOA exceeds 300 ms. At the same time, engagement (RT*_valid neutral cue_* -RT*_valid emotional cue_*) and disengagement (RT*_invalid emotional cue_*-RT*_invalid neutral cue_*) provide measures of attentional capture and attentional holding for emotional stimuli, respectively, of which a positive score of aengagement indicates an enhanced attentional capture by emotional cues while a positive score of disengagement indicates a strong attentional holding by emotional cue [48].

In this study, the spatial cueing task comprised 270 randomly presented trails. Participants viewed the fixation point for 300 ms, then one cueing facial expression was appeared on the left or right side of screen for 500 ms, and a horizontal arrow was presented immediately in one side of the screen after the cue facial expression was disappeared. The subjects pressed a key indicating where the arrow was located. When an arrow was appeared in the right side of the screen, the participants pressed the “Alt” key with forefinger of right hand; otherwise they pressed the “Ctr” key with forefinger of left hand. The horizontal arrow would disappear in 2000 ms if the subjects did not make response. Moreover, there was a 300 ms interval between the two trials, in which a fixation point was presented.

Attentional bias is comprised of facilitated orienting, disengaging and attentional avoidance. Each of the components is related to the occurrence of attentional bias at different processes of attention. Thus, measurement of these components necessitates a task that can differentiate the components. The spatial cueing task, as comparing to the other paradigms such as dot probe task and modified stroop task, has an outstanding advantage to revealing these components. Cue validity, engagement and disengagement measured using spatial cueing task provide valid measures of attentional avoidance, facilitated orienting and disengagement, respectively [47]. In contrast, the dot probe task and the stroop task can't distinguish these components and reveal the origins of attentional bias. However, the attentional bias assessed using spatial cueing task is easily influenced by the sequence effects[53]. To diminish this effect, we displayed a 300 ms inter- trail interval in the task.

In the trails, the photographs were presented in the left or right fields with an equal number of presentations. The program was compiled by using DMDX display software[54]. The display software (version number: 3.2.6.4) was set up on the computer with video card at 640×480 with 16 bits per pixel.

### Genotyping

Genomic DNA was extracted from hair follicle cells by using Chelex-100 method [55]. *COMT* Val158Met was amplified by polymerase chain reaction (PCR) by using upstream primer, 5′-CCAGCGGATGGTGGATTTCGCACGC-3′ and the downstream primer 5′-TGGGGGGGTCTTTCCTCAGCC-3′. The AC in the upstream primer was a site-directed mutagenesis for introducing a restriction site for MluI. The PCR was performed with a volume of 5 ul system containing 2.50 ul reaction MIX (Golden Easy PCR System, TIANGEN), 0.50 ul DNA template, 2.50 ul ddH2O, 0.25 ul (25 pmol/ul) upstream primer, and 0.25 ul (25 pmol/ul) downstream primer. A product of 206 bp was amplified with an initial 4 min denaturation at 94°C, followed by 30 cycles of 94°C for 30 s, 63.5°C for 30 seconds, 72°C for 30 s, and a final extension at 72°C for 3 min. The PCR product was incubated with MluI (FERMENTAS, MBI) at 37°C overnight. According to the provided protocols, the 5.0 ul incubation system contained 1.5 ul PCR products, 4.0 U MluI (10 U/ul), 0.4 ul R buffer, and 3.1 uL ddH2O. The digested mixture was analyzed by using 8% polyacrylamide gel electrophoresis with 200 V for 1.5 h, which was followed by silver staining. Finally, the genotypes were scanned by using the Bio-imaging System.

*DRD2* TaqIA was amplified by PCR. The upstream primer, 5′- ATGCCCTGCTTTCGG -3″ and the downstream primer, 5′- GAGTGTCATCAACCTCCTA -3′ were recruited. A 201 bp product was amplified with an initial 7 min denaturation at 94°C, followed by 32 cycles of 94°C for 1 min, 62°C for 30 s, 72°C for 30 s, and a final extension at 72°C for 7 min. The PCR product was incubated with TaqI at 65°C for 5 h. The incubation was performed in a volume of 5 ul system containing 1.0 ul PCR products, 3.5 U TaqI (10 U/ul), and 0.35 ul Tango™ buffer, and 3.3 ul ddH2O. The digested mixture was resolved on 8% polyacrylamide gel electrophoresis with 250 V for 1 h, which was followed by silver staining.

### Statistical Analysis

Hardy-Weinberg equilibrium tests were carried out with Finetti software. The independent samples *t* tests were conducted to examine the effects of *COMT* Val158Met on attentional bias (Met/Met and Met/Val genotypes were combined as one group because of the rare frequency of Met/Met (5.59%), which could enhance the power and reliability of the genetic behavioral study). One-way analysis of variances (ANOVAs) was performed to assess the effects of *DRD2* TaqIA on the attentional bias. The statistical power was evaluated with the G*Power program [56]. Statistical significance was referred to as *P*<0.05.

## Results

Fifteen subjects, who made more 10% errors, were excluded from our further study. The mean (410.40 ms) and standard deviation (101.31 ms) of RTs of 30 randomly selected subjects were established after the odd values were ruled out. According to the mean and standard deviation, we excluded error responses, RTs of less than 200 ms or above three standard deviations from the original data. Moreover, there were no significant gender differences in the indices of attentional biases in the present study.

The genotyping was carried out for 650 participants. Six hundred and forty four subjects were genotyped successfully at *COMT* Val158Met (Met/Met = 36, Met/Val = 231, Val/Val = 377) and 632 subjects were genotyped successfully at *DRD2* TaqIA (TT = 92, TC = 317, CC = 223). The results indicated that the two genetic variations showed no deviations from Hardy–Weinberg equilibrium (*χ* ^2^ = 0.01, *P* = 0.94; *χ* ^2^ = 1.47, *P* = 0.23).

The independent samples *t* tests indicated that *COMT* Val158Met was associated with engagement for negative expressions and the 267 individuals with Met allele (Met/Met & Val/Met) showed larger engagement bias for negative emotional content expressions than the 377 Val/Val homozygotes (3.94±0.88 vs. 1.31±0.77; *t*_(642)_ = 2.24, *P* = 0.03). The effect size indicated that this polymorphism could explain 1.00% (η^2^ = 0.01) variance in engagement bias for negative expressions. However, *COMT* Val158Met was not significantly associated with cue validities and disengagements. The effects of this polymorphism on attentional bias were displayed in table 1, and attentional bias scores of each genotype were shown as mean and standard error (SE).

**Table 1:** The modulations of *COMT* Val158Met on attentional bias.

Attentional bias	Met/Met (36) & Val/Met (231)	Val/Val (377)	*t* (642)	*P*
Cue validities				
Positive expressions	−15.57±1.88	−14.57±1.70	0.39	0.70
Neutral expressions	−23.74±1.81	−22.80±1.64	0.38	0.70
Negative expressions	−18.97±1.87	−18.66±1.61	0.13	0.90
Engagements				
Negative expressions	3.94±0.88	1.31±0.77	2.24	0.03
Positive expressions	3.87±0.94	2.08±0.83	1.42	0.16
Disengagements				
Negative expressions	0.83±0.81	2.83±0.73	1.81	0.07
Positive expressions	4.30±0.98	6.15±0.89	1.39	0.17

ANOVAs showed that *DRD2* TaqIA was significantly associated with attentional engagement bias for positive emotional expressions (*F*_(2,629)_ = 5.81, *P* = 0.003). The 92 individuals with TT genotype of TaqIA (6.92±1.60) showed much engagement bias for positive emotional content expressions than the 223 individuals with CC genotype (0.42±1.00). The effect size indicated that this polymorphism could explain 2.00% (η^2^ = 0.02) variance in engagement for positive emotional expressions. However, TaqIA was not significantly associated with cue validities and disengagements. The effects of polymorphism on attentional bias were displayed in table 2.

**Table 2:** The modulations of *DRD2* TaqIA on attentional bias.

Attentional bias	TT (92)	TC (317)	CC (223)	*F* (2,629)	*P*
Cue validities					
Positive expressions	−10.92±4.06	−15.36±1.75	−14.63±2.07	0.66	0.52
Neutral expressions	−25.31±3.51	−23.70±1.64	−20.39±2.07	1.12	0.33
Negative expressions	−20.86±3.87	−18.85±1.66	−17.30±2.02	0.44	0.65
Engagements					
Negative expressions	2.04±1.48	2.85±0.76	1.52±1.10	0.55	0.58
Positive expressions	6.92±1.60	3.42±0.91	0.42±1.00	5.81	0.003
Disengagements					
Negative expressions	2.41±1.52	1.62±0.80	1.58±0.91	0.13	0.88
Positive expressions	7.47±1.58	4.92±0.90	5.34±1.22	0.81	0.45

We further examined the interactions of *COMT* Val15*8M*et and *DRD2* TaqIA on attentional bias, in which *COMT* (Met/Met & Met/Val were combined as one group for the lower frequency of Met/Met) was an upstream gene of *DRD2* according to their roles in dopamine metabolism and signaling. The results indicated that the two genes exerted significant interactions on engagements for negative and positive emotional expressions (*F*_(5,611)_ = 2.27, *P* = 0.046, η^2^ = 0.02; *F*_(5,611)_ = 3.36, *P* = 0.005, η^2^ = 0.03). Moreover, this analysis showed that the individuals with Met/Met (Met/Val) & TT combinations showed the largest engagements bias for positive expressions (8.60±3.31) and individuals with Val/Val & CC combinations showed the smallest engagements bias for negative expressions (−0.97±1.48). The interactions of *COMT* and *DRD2* on the engagements bias were displayed in table 3.

**Table 3:** The interactions of *COMT* and *DRD2* on the engagements bias.

COMT	DRD2	Frequency	Negative expressions	Positive expressions
Met/Met (Val/Met)	TT	26	1.36±2.28	8.60±3.31
	TC	132	4.11±1.21	3.67±1.34
	CC	96	4.95±1.57	2.54±1.34
	Total	254	4.14±0.89	3.74±0.93
Val/Val	TT	58	2.58±1.96	5.89±1.86
	TC	186	2.04±1.01	2.86±1.28
	CC	119	−0.97±1.48	−1.64±1.47
	Total	363	1.14±0.78	1.87±0.88

A power analysis was implemented by using the G*Power program. The sample size revealed more than 95% power for the detection of significant associations (*P*<0.05), when the tested variations had a medium genetic effects, under an effect size index of 0.30 [57].

## Discussion

The study investigated the modulations of *COMT* and *DRD2* on attentional bias for emotional facial expressions in a college student population. The results demonstrated that *COMT* Val158Met underpinned the individual difference in attentional bias for negative emotional expressions, whereas *DRD2* TaqIA underpinned the individual difference in attentional bias for positive emotional expressions.

*COMT* Val158Met was associated with the engagement, but not the disengagement for negative emotional expressions. The individuals with Met allele showed more engagement bias for negative emotional expressions. These findings implicated that Val158Met regulated the enhanced attentional capture by negative emotional expressions and the individuals with Met allele was more liable to be attracted by the negative emotional stimuli. We thought that the high sensitivity to unpleasant stimuli of the Met carriers might be a result of more engagement bias for negative emotional expressions [26]. Moreover, studies has evidenced that anxiety and depression-linked attentional bias is characterized at selective processing of negative stimuli [58], [59], [60]. Therefore, this attentional bias can lead to an increased perception in negative stimuli that were accompanied by more frequencies of anxiety. As a feedback, the increased perception in negative stimuli resulted in a high risk of individuals with Met allele in anxiety [61].

*DRD2* TaqIA was associated with engagement for positive emotional content of expressions, and the individuals with TT genotype of TaqIA showed more engagement bias for positive emotional expressions. These findings implicated that the attention of individuals with TT genotype was liable to be captured by positive emotional stimuli. So far, previous studies have shown the T allele is a genetic marker of reward sensitivity [28] and the individuals with T allele always favor the reward-related cues such as palatable food, tobacco and heroin because these cues can decrease the negative feelings by activating the release of brain dopamine [62]. Positive facial expressions could be considered as reward related-cues which could induce the participants' pleasant experiences. As a feedback, they were able to capture more attention resources.

Evidence from psychological studied has revealed that attentional bias is related to the different processes of attention [48], [63]. The engagement expresses the bias of early vigilance while disengagement denotes the bias of later attention holding [64]. In this study, *COMT* and *DRD2* were both associated with engagement bias, but not disengagement bias. The results indicated that *COMT* and *DRD2* were involved in the vigilance of emotional stimuli, and Met allele of *COMT* facilitated the vigilance bias for negative facial expressions while T allele of TaqIA facilitated the vigilance bias for positive facial expressions.

In the study, using the single genetic loci model, *COMT* and *DRD2* showed selective main effects on the attentional bias for negative and positive emotional expressions, respectively. This finding suggests that there was a significant difference in the genetic foundation of attentional bias between positive and negative stimuli although the T allele of TaqIA increased the release of dopamine [32], [34], [35] and Met allele of *COMT* leaded to an increased in dopamine levels of in the synaptic clef [24], [25]. Moreover, in the present study, *COMT* Val158Met and *DRD2* TaqIA only accounted for 1.00% and 2.00% variances of attentional bias for facial expressions. This genetic power approximately equaled to the previous results which indicated a certain single genetic loci could explain subtle a variance in attentional bias [49], [65], [66], [67], [68]. The results further indicated that attentional bias was a polygenetic trait and each of the genes exerts a subtle effect on the individual difference although it has a substantial heritability [57]. *COMT* and *DRD2*, as well as *5-HTTLPR*, *ADRA2B*, *DBH* and *MAOA* in previous studies [49], [65], [66], [68], were a small part of the candidate genes underlying the individual differences of attentional bias because of the complex phenotype including many components such as facilitated engagement, delayed disengagement, attentional controlling and emotional regulation [47]. Therefore, more genetic loci in dopaminergic and serotoninergic systems should be selected in the future investigation.

*DRD2* TaqIA and *COMT* Val158Met are both related to the dopamine levels of the synaptic clef. Consistent with the expected results, the study indicated that the two genes exerted significant interactions on the attentional engagements for negative and positive expressions. The interactive effects could explain 2.00% and 3.00% variances in engagements for negative and positive emotional expressions, respectively. This finding provided strong evidence of the endogenous interactions between *COMT* and *DRD2* on attention bias. Liking many gene-gene interaction studies, the interactions of two genes exceeded their main effects. The interesting results further indicated that attentional bias was multiple genes expressed phenotype and the interactions of the genes exerted more contributions to the individual difference and the substantial heritability of attentional bias [57]. However, a larger sample size was needed in gene-gene interaction analysis. In the research, the lower frequency of Met/Met was limitation in exploring the interactions. Therefore, further research is needed to replicate these findings.

Among the interactional effects, we also observed that Met/Met (Met/Val) & TT combinations exerted the largest impact on engagements bias for positive expressions while Met/Met (Met/Val) & CC combinations exerted the largest impact on engagements bias for negative expressions. These interesting results implicated that there was a difference in the patters of *COMT* and *DRD2* interacting on positive and negative expressions. In the framework of *COMT* and *DRD2* interacting on negative facial expressions, the interactions of Met/Met (Met/Val) & TT combinations and Val/Val & CC combinations showed the smallest impact on engagements bias while the interactions of Met/Met (Met/Val) & CC showed the largest impact on engagements bias. Thus, we arrived at a view that the individuals (Met/Met (Met/Val) & TT) with the lower dopamine levels and individuals (Val/Val & CC) with the higher dopamine levels showed smallest engagements bias, while the individuals (Met/Met (Met/Val) & CC) with the moderate dopamine levels showed the largest engagements bias because T allele of TaqIA and Met allele of *COMT* were related to the increased in dopamine levels of in the synaptic clef. These findings implicated that there was an inverted U-shaped dose-effect curve between dopamine levels [69], [70] and attentional bias for negative facial expressions. However, in the framework of *COMT* and *DRD2* interacting on engagements bias for positive facial expressions, the effect of dopamine levels on engagements bias did not display the inverted U dose-effect curve. The Met/Met (Met/Val) & TT combinations with higher dopamine levels showed the largest impact, the Val/Val & CC combinations with lower dopamine levels showed the smallest impact, while Met/Met (Met/Val) & CC and Val/Val & TT combinations with moderate level dopamine levels exerted the moderate impacts.

Three limitations of this study need to be mentioned. Firstly, given the wide distribution of the sample, we could not exclude the potential population admixture effect although the large sample size could minimize the distortion. Secondly, only self-report scales were used to screen for neurological and psychiatric disorders of this college sample. Therefore, future research should employ an objective systematic tool. Thirdly, the adjusting for multiple testing was not performed, thus more work was needed to examine the results. However, this study has implications for understanding the genetic contributions of dopamine to cognitive bias.

## Conclusions

A population-based study was performed to investigate the modulations of *COMT* and *DRD2* on attentional bias for emotional facial expressions. We observed that *COMT* Val158Met and *DRD2* TaqIA were associated with engagement for negative and positive facial expressions, respectively. The results suggest the inter-subject differences in attentional bias for emotional facial expressions are partially modulated by some functional polymorphisms in dopaminergic genes.
